# Association between sleep quality and MCI in older adult patients with multimorbidity

**DOI:** 10.3389/fpubh.2025.1547425

**Published:** 2025-03-18

**Authors:** Ting Yang, Guoyan Zheng, Shuzhi Peng

**Affiliations:** ^1^School of Nursing, Sichuan Vocational College of Health and Rehabilitation, Zigong, China; ^2^College of Health Management, Shanghai Jian Qiao University, Shanghai, China

**Keywords:** multimorbidity, RCS, sleep quality, MCI, PSQI

## Abstract

**Objective:**

To explore the relationship between sleep quality and mild cognitive impairment in older adult patients with multimorbidity.

**Methods:**

The general data of older adult patients with chronic diseases were collected, and the sleep quality and mild cognitive impairment (MCI) of older adult patients with multimorbidity were investigated by questionnaire. Logistic regression model and restricted cubic spline (RCS) model were used to analyze the correlation between sleep quality and MCI in older adult patients with multimorbidity.

**Results:**

There are 902 valid samples in this study, of which 333 (36.9%) have MCI. The number of chronic diseases ranges from 2 to 6, and the number of types of medication ranges from 0 to 7. The score of PSQI is 2–18, with an average score of 11.13. MoCA score range is 7–30. The MoCA score of MCI patients is lower than that of Non-MCI patients. In all three models, PSQI score is significantly correlated with MCI. The results of the segmented regression analysis show that: the inflection point of MCI’s PSQI scoring relationship is 12. RCS result display: with the increase of PSQI score, the OR increases between PSQI score and MCI, when PSQI score reaches 12, OR is significantly higher than 1.

**Conclusion:**

Sleep quality is an important influencing factor of MCI, and there is a threshold effect in the above association. According to this correlation, health professionals can take measures to improve the sleep quality of older adult patients with multimorbidity to reduce the occurrence of MCI.

## Introduction

In 2020, the total number of older adult people aged 60 and above in Chinese mainland will be 264 million, accounting for 18.7% of the total population (1). With the aggravation of population aging, the number of older adult patients with chronic diseases has also increased year by year, and the coexistence of two or more chronic diseases is more common. There are many older adult patients with multimorbidity in China who have Mild cognitive impairment (MCI) and sleep disorders (2). Investigation shows that about 50–60% of the older adult in China complain about poor sleep. The prevalence of MCI in the older adult over 60 years old is estimated to be 15.5% (3). Most patients regard MCI symptoms and sleep problems as a normal aging process. Because of insufficient understanding of the disease, they usually refuse to see a doctor.

Multimorbidity means that a person has two or more chronic health problems at the same time (4). MCI means that the decline of cognitive ability is greater than the expected degree of individual age and education level, but it has no obvious influence on daily life activities (5). Due to the decline of physical function, the older adult with multimorbidity have changed their sleep structure and circadian rhythm, which makes them more likely to cause sleep quality decline and even sleep disorders (6). In recent years, it is considered that sleep is a controllable risk factor for MCI (7). Sleep changes may be a valuable sign of early MCI (8).

At present, many studies have found that the decline of sleep quality and the occurrence and development of MCI are vicious cycles (9). It is difficult for older adult patients with multimorbidity to have a good and deep sleep when they have symptoms related to diseases (10). Sleep problems can lead to or aggravate the occurrence and development of MCI, and even develop into Alzheimer’s disease (11). Therefore, it is of great practical significance to deeply understand the sleep situation of older adult patients with multimorbidity, and to give targeted prevention and early intervention, so as to reduce the influence of MCI on the development of chronic diseases of the older adult and alleviate the medical burden brought by the rapid increase of social medical needs of older adult patients.

In recent years, there have been many studies on the relationship between sleep problems and cognitive dysfunction in older adult patients with chronic diseases, but there are few studies on the relationship between sleep problems and MCI in older adult patients with multimorbidity. Therefore, in our study, we use Pittsburgh Sleep Quality ScaleI (PSQI) and Montreal Cognitive Assessment Scale (MoCA) to evaluate the sleep quality and MCI of older adult patients with multimorbidity. To study the potential correlation between sleep quality and MCI in order to control the development of MCI into Alzheimer’s disease by controlling sleep quality. In the past, researchers liked to study the sleep problems and cognitive dysfunction of older adult patients with chronic diseases separately, and included them as classified variables in the model analysis (12–15). This will lose some information as a continuous variable. At present, there is little research on the relationship between PSQI score as a continuous variable and MCI risk. In this study, the sleep quality and MCI of older adult patients with chronic diseases in Shanghai and Sichuan were investigated, and the survey data were analyzed to explore the relationship between PSQI score and MCI through RCS model.

## Materials and methods

### Participants

From July 2024 to September 2024, older adult patients with chronic diseases were collected in hospitals in Sichuan and Shanghai. The study included both inpatients and outpatients. Inclusion criteria: ①Age ≥ 60 years old; ② Adapting to multimorbidity (two or more coexisting diseases, including but not limited to hypertension, diabetes, cardiovascular disease, chronic obstructive pulmonary disease (COPD), and arthritis); Exclusion criteria: ① Age < 60 years old; ② Severe mental disorders; ③ Severe visual and hearing impairment; ④Diseases of central nervous system (e.g., stroke, Parkinson’s disease). All participants knew the purpose of the survey and signed an agreement authorizing the collection of data.

### Instruments and measurements

General information questionnaire was used to investigate and test the basic characteristics of participants, such as sex, age, BMI, marital status, education attendance and so on. Sleep quality was evaluated by Pittsburgh Sleep Quality Index (PSQI). PSQI can evaluate the sleep quality of patients within 1 month, including the overall score and seven component scores (16). The component items are as follows: subjective sleep quality, sleep latency, sleep duration, sleep efficiency, sleep disorders, use of sleep drugs and daytime dysfunction (17). The score range of each part is 0–3, with a perfect score of 21. The higher the score, the worse the sleep quality (18). Cognitive function was assessed by Montreal cognitive assessment (MoCA) (19). The main scoring items of MoCA scale include naming, attention, language, abstraction, delayed recall, orientation, visual space and executive function (20). Divide normal and MCI with 26 as the demarcation value, score ≥ 26 is Non-MCI, and MCI means score < 26 (21).

### Sampling method

**Figure 1:**
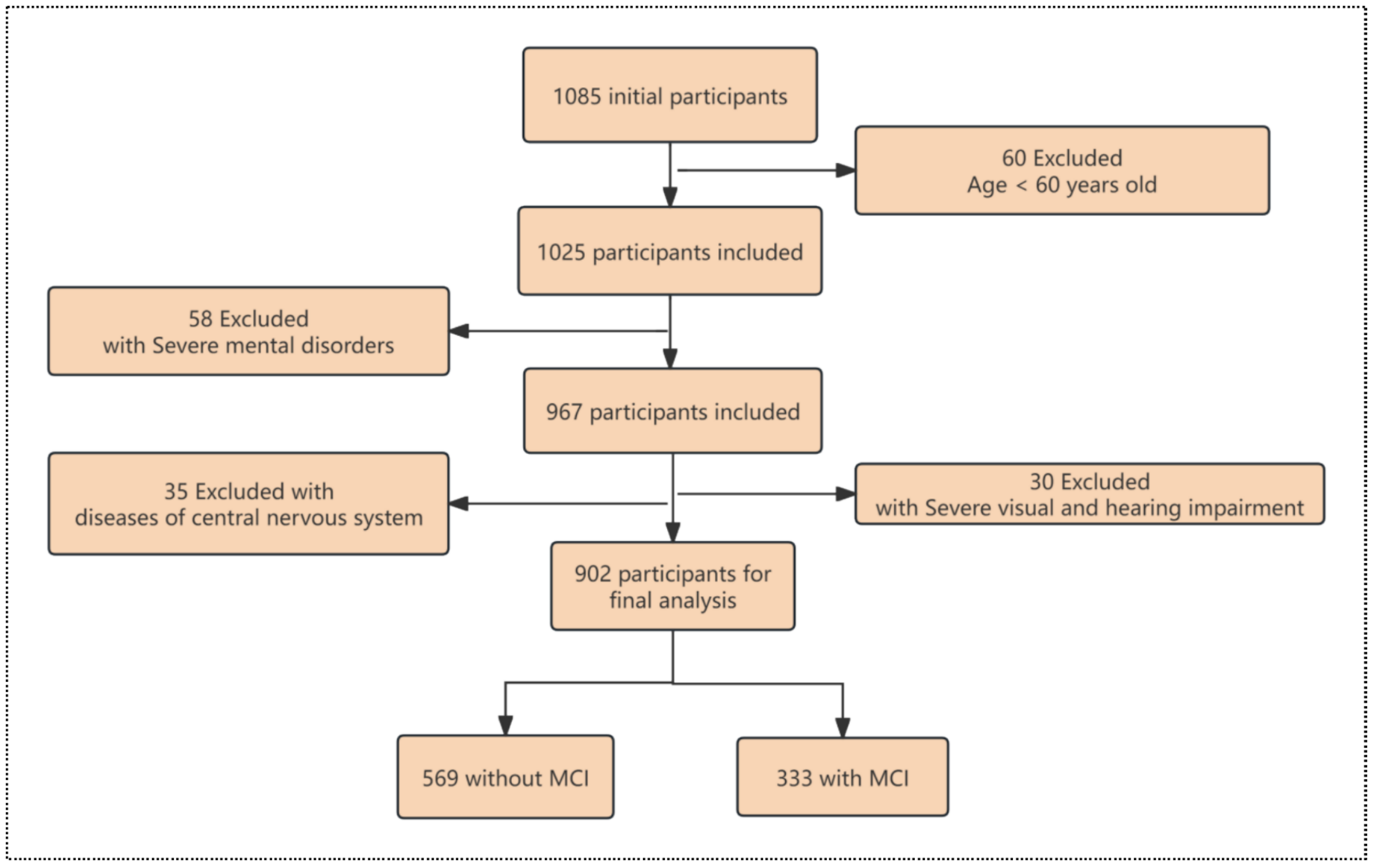
The flow chart of sample collection.

A questionnaire survey was conducted in Sichuan and Shanghai from June 2024 to July 2024. Investigators have received unified training. In order to ensure that investigators describe and explain the contents of the investigation in the same way, all our investigators are the same group of uniformly trained personnel. The sample size was determined using sociological research methods and calculated using the following formula: n = p × z^2^ × (1 – p)/e^2^, where prefers to the overall proportion, z refers to the confdence coefcient, and e refers to the allowable error (22). If *p* = 0.8, the maximum variance could obtain a relatively conservative sample size. At the same time, the allowable error of 5–10% and the placing interval of 95% were selected in this survey, the calculated available sample size should be at least 683. See Figure 1 for the sample collection flow chart.

### Statistical analysis

Participants were divided into two groups according to whether they had MCI. All continuous variables were tested for normality using the Shapiro–Wilk test. Variables that did not meet the normality assumption were log-transformed. For weighted feature description, continuous variables were presented as means ± standard error (SE) and categorical variables were presented by percentage (%) (23). The difference of baseline variables was tested by t test and chi-square test. Using logistic regression model, the odds ratio (OR) and 95% confidence interval (CI) of the correlation between Sleep quality and MCI were discussed. Model 1, no covariate was adjusted. Model 2 was adjusted for sex, age, BMI, marital status, education attainment, living conditions, social contact, exercise, smoking, drinking. Model 3 added number of chronic diseases, number of types of medication, PSQI score, subjective sleep quality, sleep latency, sleep duration, sleep efficiency, sleep disorders, use of sleep drugs and daytime dysfunction in model 2. RCS is used to explore nonlinear problems (24). In addition, we also conducted subgroup analysis to investigate whether this association was changed by social demography, disease-related conditions or lifestyle characteristics in the fully adjusted model (25). Then a two-segment linear regression model is established to calculate the turning point. All statistical analysis was carried out by R 4.3.3 software, and *p*-values<0.05 was considered to be statistically significant.

## Results

There are 902 valid samples in this study, of which 333 (36.9%) have MCI. The age range of all participants is 60–91 years old, with an average age of 67.76 years old. There are more men than women with MCI, and there are more people with low education than those with high education. Among the related factors of diseases, the number of chronic diseases ranges from 2 to 6, and the number of types of medication ranges from 0 to 7. The score of PSQI is 2–18, with an average score of 11.13. The scores of seven components of sleep quality and the occurrence of MCI were analyzed by t test, and it was found that there was significant difference between each component and the occurrence of MCI. MoCA score range is 7–30. The MoCA score of MCI patients is lower than that of Non-MCI patients (See Table 1 for details).

**Table 1:** Demographic characteristics and disease-related factors of the study population.

Variables	All participants *n* = 902	Non-MCI *n* = 569	MCI *n* = 333	*P*-value
Sex				0.889
Male	514 (56.98)	323 (35.81)	191 (21.18)	
Female	388 (43.02)	246 (27.27)	142 (15.74)	
Age (years)	67.76 (43.02)	66.31 (4.90)	70.23 (7.09)	<0.01
BMI (kg/m^2^)	24.30 (2.17)	24.37 (2.06)	24.19 (2.33)	0.234
Marital status				<0.01
Married	640 (70.95)	424 (47.01)	216 (23.95)	
Never married	43 (4.77)	33 (3.67)	10 (1.11)	
Widowed/divorced	219 (24.28)	112 (12.42)	107 (11.86)	
Education attainment				<0.01
Below high school	601 (66.63)	315 (34.92)	286 (31.71)	
High school	145 (16.08)	118 (13.08)	27 (2.99)	
College or above	156 (17.29)	136 (15.08)	20 (2.22)	
Living conditions				<0.01
Living alone	169 (18.74)	103 (11.42)	66 (7.32)	
Live with family	711 (78.82)	461 (51.11)	250 (27.72)	
Living with caregivers	22 (2.44)	5 (0.55)	17 (1.88)	
Social contact				<0.01
Every day	146 (16.19)	107 (11.86)	39 (4.32)	
5–6 times a week	178 (19.73)	110 (12.20)	68 (7.54)	
3–4 times a week	363 (40.24)	223 (24.72)	140 (15.52)	
1–2 times a week	186 (20.62)	122 (13.53)	64 (7.10)	
Never	29 (3.22)	7 (0.78)	22 (2.44)	
Exercise				<0.01
Every day	206 (22.84)	152 (16.85)	54 (5.99)	
5–6 times a week	176 (19.51)	113 (12.53)	63 (6.98)	
3–4 times a week	360 (39.91)	222 (24.61)	138 (15.30)	
1–2 times a week	113 (12.53)	72 (7.98)	41 (4.55)	
Never	47 (5.21)	10 (1.11)	37 (4.10)	
Smoking				0.005
Never smoking	553 (61.31)	368 (40.80)	185 (20.51)	
Current smoking	249 (27.61)	136 (15.08)	113 (12.53)	
Former smoking	100 (11.09)	65 (7.21)	35 (3.88)	
Drinking				<0.01
Never drinking	535 (59.31)	331 (36.70)	204 (22.62)	
Current drinking	241 (26.72)	139 (15.41)	102 (11.31)	
Former drinking	126 (13.97)	99 (10.98)	27 (2.99)	
Number of chronic diseases	3.56 (1.17)	3.20 (1.15)	4.18 (0.94)	<0.01
Number of types of medication	1.84 (1.50)	1.33 (1.31)	2.72 (1.40)	<0.01
PSQI score	11.13 (2.53)	10.37 (2.64)	12.42 (1.67)	<0.01
Subjective sleep quality	2.05 (0.53)	1.98 (0.38)	2.18 (0.70)	<0.01
Sleep latency	2.17 (0.48)	2.06 (0.44)	2.37 (0.50)	<0.01
Sleep duration	2.02 (0.41)	1.95 (0.39)	2.15 (0.42)	<0.01
Sleep efficiency	1.94 (0.52)	1.86 (0.54)	2.08 (0.44)	<0.01
Sleep disorders	1.45 (0.78)	1.26 (0.84)	1.79 (0.52)	<0.01
Use of sleep drugs	0.16 (0.39)	0.07 (0.26)	0.32 (0.51)	<0.01
Daytime dysfunction	1.34 (0.84)	1.14 (0.91)	1.67 (0.60)	<0.01
MoCA score	25.54 (1.87)	26.62 (0.88)	23.70 (1.66)	<0.01

Data are presented as mean (SD) or *n* (%).

**Figure 2:**
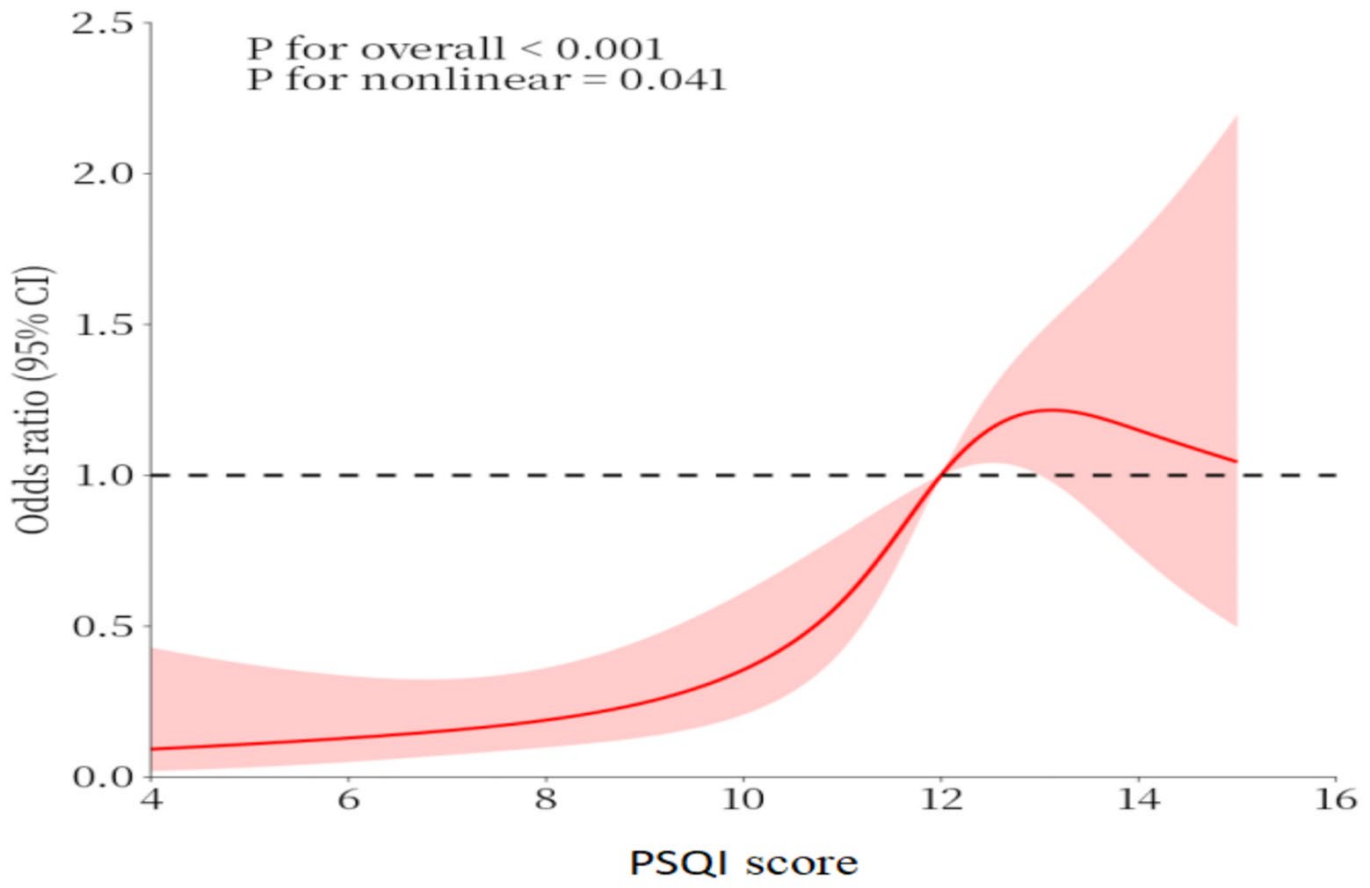
The dose–response relationship between PSQI score and MCI.

In all three models, PSQI score is significantly correlated with MCI (see Table 2 for details). Considering the significant relationship between PSQI score and MCI, we conducted a piecewise regression analysis (see Table 3 for details). The inflection point of MCI’s PSQI scoring relationship is 12. On this basis, we adopt a cubic spline model based on model 3 (see Figure 2). With the increase of PSQI score, the OR of correlation between PSQI score and MCI increases. When PSQI score reaches 12, OR is significantly higher than 1.

**Table 2:** Logistic regression results of associations between sleep quality and MCI.

Model	OR	95%CI	*P*-value
Model 1			
PSQI score	1.596	(1.464, 1.741)	<0.001
Model 2			
PSQI score	1.607	(1.460, 1.768)	<0.001
Model 3			
PSQI score	1.307	(1.172, 1.458)	<0.001

Model 1, no covariate was adjusted. Model 2, sex, age, BMI, marital status, education attainment, living conditions, social contact, exercise, smoking, drinking were adjusted. Model 3, sex, age, BMI, marital status, education attainment, living conditions, social contact, exercise, smoking, drinking, number of chronic diseases, number of types of medication, PSQI score, subjective sleep quality, sleep latency, sleep duration, sleep efficiency, sleep disorders, use of sleep drugs and daytime dysfunction were adjusted.

**Table 3:** Threshold effect analysis between sleep quality and MCI.

Outcome	OR (95%CI)	*P*-value
Two-piecewise linear regression model		
PSQI score < 12	1.434 (1.032–1.993)	0.032
PSQI score ≥ 12	1.171 (0.926–1.479)	0.187
Log-likelihood ratio test		<0.001

## Discussions

### Present situation of MCI in older adult patients with multimorbidity

A total of 902 older adult patients with multimorbidity were included in this study. The age range of all participants was 60–91 years, with an average age of 67.76 years. The number of chronic diseases ranged from 2 to 6 with an average of 3.56. The possible reasons are as follows: the pathological states of various chronic diseases in the older adult will affect each other, and the interweaving of various chronic diseases will lead to the aggravation of brain ischemia and hypoxia and form a vicious circle (26).

### Relationship between sleep quality and MCI

In this study, the total score of PSQI should be used as the overall evaluation of sleep quality. Our study found that PSQI score is positively correlated with the OR of MCI. The score of MCI group is significantly higher than that of non-MCI group. After adjusting the related confounding factors, it is still found that the PSQI score is high, and the risk of MCI in older adult patients with multimorbidity is higher. This is consistent with the research results of McKinnon et al. (27). Some studies have pointed out that the increase of serum cortisol and corticotropin-releasing hormone is related to the decline of cognitive ability (28). However, the levels of serum cortisol and corticotropin-releasing hormone increased in the older adult population with decreased sleep quality (29).

Mayer et al. (7) used PSQI to evaluate sleep quality and analyze the relationship between sleep disorder and MCI. However, in their study, the PSQI score was divided into five groups, which were divided into sleep disorder group and normal sleep group. In our research, PSQI is regarded as a continuous variable, and the RCS model is adopted. It is found that the score of PSQI has a threshold, and subgroup analysis further confirms these results. When PSQI score reaches 12, OR is significantly higher than 1. This shows that when the PSQI score is higher than 12, it is the risk factor for cognitive decline. Higher threshold may reflect the compounded effects of multimorbidity on sleep quality and cognitive function. The lack of association in patients with severe sleep disorders (PSQI≥12) may be due to ceiling effects or the presence of other confounding factors that were not fully accounted for in the analysis.

Sleep is a changeable risk factor. Based on the results of this study, it is found that the problem of sleep quality should be taken into account when formulating intervention measures for MCI in the older adult with multimorbidity. Keeping good sleep behavior, reducing sleep latency, improving sleep efficiency and improving daytime sleepiness may be one of the measures to prevent MCI in the older adult with multimorbidity or delay the development of MCI into dementia.

### Strengths and limitations

Previous studies mostly discussed the relationship between sleep quality and MCI by classified variables, or the linear relationship between PSQI score and MoCA score, which limited the comprehensive interpretation of the relationship between PSQI and MCI abnormal illness (9, 27, 30), while the RCS model is a kind of nonlinear regression model, which can capture the nonlinear relationship between independent variables and dependent variables more flexibly and has good explanatory power (24). MCI is a reversible or potentially reversible clinical syndrome (31). Early identification and effective intervention are helpful to reverse the occurrence of MCI.

The sample size of this study is 902 cases, which cannot represent all the older adult people with comorbidity, and more centers and larger samples are needed in the future. Although the inclusion of exclusion criteria and statistical models controlled some confounding factors, the results were still influenced by unmeasured or not excluded interference factors, and related factors will be further strictly controlled in future research. This study is a cross-sectional study. Prospective cohort study is needed to further clarify the causal relationship between sleep quality and MCI in older adult with multimorbidity.

## Conclusion

Our study found that there is a nonlinear correlation between PSQI score and MCI in the older adult with multimorbidity after adjusting for potential confounding factors. Sleep quality is an important influencing factor of MCI, and there is a threshold effect in the above association. According to this correlation, health professionals can take measures to improve the sleep quality of older adult patients with multimorbidity to reduce the occurrence of MCI. In addition, the evidence of this study also encourages older adult patients with multimorbidity to evaluate their sleep quality and reduce the occurrence of MCI.

## Data Availability

The original contributions presented in the study are included in the article/supplementary material, further inquiries can be directed to the corresponding author.
